# Enhancement of Tumour-Specific Immune Responses *In Vivo* by ‘MHC Loading-Enhancer’ (MLE)

**DOI:** 10.1371/journal.pone.0006811

**Published:** 2009-09-07

**Authors:** Katharina Dickhaut, Sabine Hoepner, Jamina Eckhard, Karl-Heinz Wiesmueller, Luise Schindler, Guenther Jung, Kirsten Falk, Olaf Roetzschke

**Affiliations:** 1 Max-Delbrück-Center for Molecular Medicine (MDC), Berlin, Germany; 2 Charité, Berlin, Germany; 3 Singapore Immunology Network (SIgN), Agency for Science, Technology and Research (A*STAR), Biopolis, Singapore; 4 EMC microcollections GmbH, Tübingen, Germany; 5 Eberhard-Karls University, Tübingen, Germany; New York University School of Medicine, United States of America

## Abstract

**Background:**

Class II MHC molecules (MHC II) are cell surface receptors displaying short protein fragments for the surveillance by CD4+ T cells. Antigens therefore have to be loaded onto this receptor in order to induce productive immune responses. On the cell surface, most MHC II molecules are either occupied by ligands or their binding cleft has been blocked by the acquisition of a non-receptive state. Direct loading with antigens, as required during peptide vaccinations, is therefore hindered.

**Principal Findings:**

Here we show, that the *in vivo* response of CD4+ T cells can be improved, when the antigens are administered together with ‘MHC-loading enhancer’ (MLE). MLE are small catalytic compounds able to open up the MHC binding site by triggering ligand-release and stabilizing the receptive state. Their enhancing effect on the immune response was demonstrated here with an antigen from the *influenza virus* and tumour associated antigens (TAA) derived from the NY-ESO-1 protein. The application of these antigens in combination with adamantane ethanol (AdEtOH), an MLE compound active on human HLA-DR molecules, significantly increased the frequency of antigen-specific CD4+ T cells in mice transgenic for the human MHC II molecule. Notably, the effect was evident only with the MLE-susceptible HLA-DR molecule and not with murine MHC II molecules non-susceptible for the catalytic effect of the MLE.

**Conclusion:**

MLE can specifically increase the potency of a vaccine by facilitating the efficient transfer of the antigen onto the MHC molecule. They may therefore open a new way to improve vaccination efficacy and tumour-immunotherapy.

## Introduction

Since Edward Jenner discovered a small pox vaccine in 1789, investigators have focused for more than 200 years on developing strategies to improve human life by creating vaccines against various infectious diseases. Up to now, over 20 diseases are preventable due to successful vaccination. More recently, immunologist started to explore the possibility to amplify patient's immune response against tumours using vaccination. As first ‘tumour-specific’ vaccines “Gardasil” (Merck) [1] and “Cervarix” (GlaxoSmithKline) [2] have been introduced for prophylaxis against cervical cancer. Analogue to ‘traditional’ vaccines, they contain viral components of the cancer-causing human papilloma virus (HPV), whereas treatment of most other cancers may rely on targeting self-antigens. A promising group of these tumour-associated antigens (TAA) includes cancer testis antigens (CT), which are expressed on various tumours while being absent on normal tissue except for the testis [3]. They therefore represent attractive targets for tumour-immunotherapy. Among those, NY-ESO-1 is one of the most prominent and well-studied antigens [4]. Several clinical trials have been launched to determine its efficacy [5].

Vaccination efficacy depends largely on the the immune-modulatory capacity of the adjuvant, also known as ‘immunologist’s “dirty” little secret' [6]. Innate signals being co-delivered with the vaccine drastically influence the strength and direction of the immune response. The importance of microbial-derived vaccine additives, such as the TLR-agonists CpG [7] or Pam3Cys [8], [9], and the enhancing effect of cell-damage associated compounds, namely uric acid [10] and ATP [11] has just begun to be fully acknowledged and represents a field that is currently under extensive investigation [12].

Another way to increase antigenicity is to improve antigen delivery to immunogenic APC. T cell epitopes linked to antibodies targeting DC specific surface molecules such as DC-SIGN [13] or DEC-205 [14] have demonstrated their efficacy in various mouse models. While these approaches require the generation of rather large recombinant fusion proteins, the use of ‘MHC-loading enhancer’ (MLE) may represent an elegant alternative. MLE are small catalytic compounds that are simply added to the antigen mixture [15]–[20]. The mechanism is based on the fact that on the cell surface the efficacy of antigen loading is hindered by the lack of empty and accessible MHC II molecules. They are either occupied already by endogenous ligands or, upon loosing their ligand, have acquired a non-receptive state. The latter is characterized by the inability of an empty MHC molecule to bind a peptide ligand, putatively caused by a conformation in which the peptide-binding site is blocked [21]. The rapid transformation into this non-receptive state is regarded as a safety-mechanism, avoiding the unwanted and potential dangerous loading and presentation of self-antigens on the surface.

We had shown earlier that small organic MLE can enhance antigen loading by reversing the inactive state and by triggering the release of ligands with lower affinity [16], [18]. By targeting the conserved P1-pocket located within the binding cleft of MHC II molecules, these MLE are able to stabilize the peptide receptive state, which allows efficient loading of APC with T cell antigens. The interaction is highly specific. Organic MLE carrying adamantyl groups such as adamantane ethanol (AdEtOH) are effective only on allelic variants of HLA-DR expressing a glycin residue at the dimorphic position β86 [18], while the allel-specificity of catalytic amino acid derivatives (‘peptide-MLE’) depends on their active amino acid side chain [19].

Several studies in mouse models have shown that long-term protection requires an adequate and sustained CD4+ T cell response, which provides help to effector cells via the production of several cytokines [22]–[24]. Hence, also the T cell priming for tumour immune responses requires peptides loaded efficiently onto MHC II molecules. *In vitro*, the presence of MLE during antigen loading increased the sensitivity of the CD4+ T cell response for a model antigen by up to two orders of magnitude [18]. Here we show that the enhancing effect is also observed *in vivo*. When using MLE as an additive during vaccination with TAA, a significant increase in the number of activated CD4+ T cells was observed.

## Results

### MLE facilitate antigen-loading of HLA-DR expressing dendritic cells

In previous studies we could show that certain phenol- or adamantyl-derivatives can increase peptide-loading of HLA-DR molecules in a catalytic fashion [16], [18]. As ‘MHC-loading enhancer’ (MLE) they were able to act both on soluble as well as on membrane-bound MHC molecules of HLA-DR transfected fibroblast cells and EBV transformed B cells. However, with regard to vaccination and immune therapy, the most important antigen presenting cells are dendritic cells (DC). With short catalytic dipeptides we have demonstrated that also these cells are prone to induced ligand-exchange [19]. Given their potential as additives for peptide vaccination we therefore investigated the impact of organic MLE on DC. Adamantane ethanol (AdEtOH) was used as MLE model compound throughout this study [16], [18].

**Figure 1:**
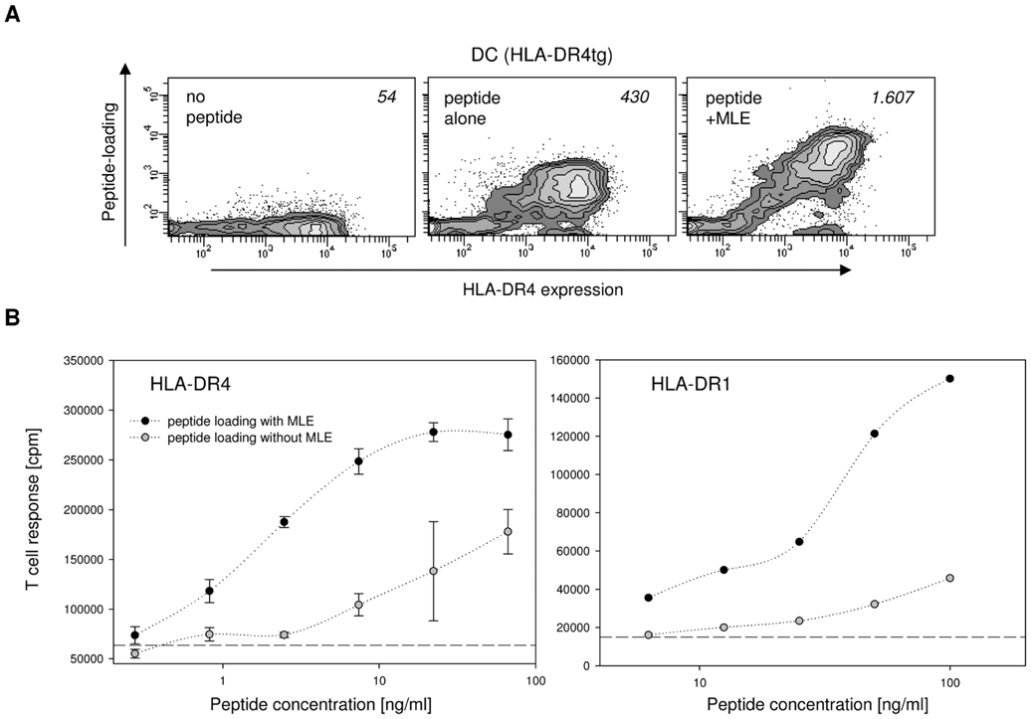
Influence of MLE on the class II MHC peptide-loading of dendritic cells. (A) Cell surface loading. HLA-DR4 expressing dendritic cells (DC) generated from the bone marrow of HLA-DR4 transgenic mice were incubated for 4 h with medium alone (left panel) or with 5 µg/ml biotinylated HA 306–318 peptide in the absence (middle panel) or presence of 250 µM AdEtOH, the model MLE compound used throughout this study (right panel). Contour plots are shown for DC after staining with anti-HLA-DR antibody (→ MHC expression) and streptavidin (→ peptide load). Mean peptide loading (MFI of streptavidin signal) is indicated. (B) CD4+ T cell response. DC from HLA-DR4tg mice (left panel) and from HLA-DR1tg mice (right panel) were pulsed for 4 h with indicated amounts of HA 306–318 peptide in the absence (open circle) and presence (closed circle) of 250 µM AdEtOH. The cells were used to challenge HA 306–318 specific, HLA-DR4-restricted 8475/94 cells and HLA-DR1-restricted EvHA/X5 T cell hybridoma cells, respectively. Background proliferation was measured in absence of peptide (dashed line).

DC derived from the bone marrow of HLA-DR4-transgenic mice were pulsed in the presence or absence of MLE with biotinylated HA 306–318 peptide (HA), an epitope derived from the hemagglutinin protein of the *influenza virus* [25]. The amount of HA peptide bound to the surface of the cells was then determined by flow cytometry, performing a double staining with HLA-DR antibody and streptavidin conjugates. As shown in Fig. 1A, peptide loading was indeed strongly enhanced when MLE were present during pulsing. Compared to the control staining without any peptide (left panel) the incubation with peptide alone revealed only a marginal increase in peptide signal (middle panel). The presence of MLE, in contrast, generated a very clear peptide-specific staining particularly on the HLA-DR^bright^ cells. The signal of bound peptide correlated directly with the HLA-DR4 expression level of the cell, confirming the specificity of the loading reaction.

To assure that improved peptide loading by MLE directly translates into an increased sensitivity of the antigen-specific T cell response, the DC were used as APC in an *in vitro* T cell proliferation assay to challenge two HA-specific CD4+ T cell hybridoma lines (Fig. 1B). As depicted in Fig. 1B, both for the HLA-DR4 (left panel) as well as HLA-DR1 expressing DC (right panel), the threshold for T cell activation could indeed be shifted by more than one order of magnitude towards lower antigen concentrations. With peptide alone the HLA-DR1 restricted EvHA T cells (right panel) responded only weakly to DC incubated at concentrations below 100 ng/ml. The addition of the MLE, however, resulted in the typical sigmoidal dose response curve typically observed only at higher antigen concentration. This effect was even more pronounced for the HLA-DR4 restricted 8475/94 T cells which strongly proliferated in response to antigen concentrations below 10 ng/ml when MLE was present during peptide loading (right panel). Thus, MLE can significantly improve the antigen-specific CD4+ T cell response by increasing the amount of MHC II/antigen complexes on the cell surface of DC.

### Selective enhancement of CD4+ T cell responses in vivo by MLE

**Figure 2:**
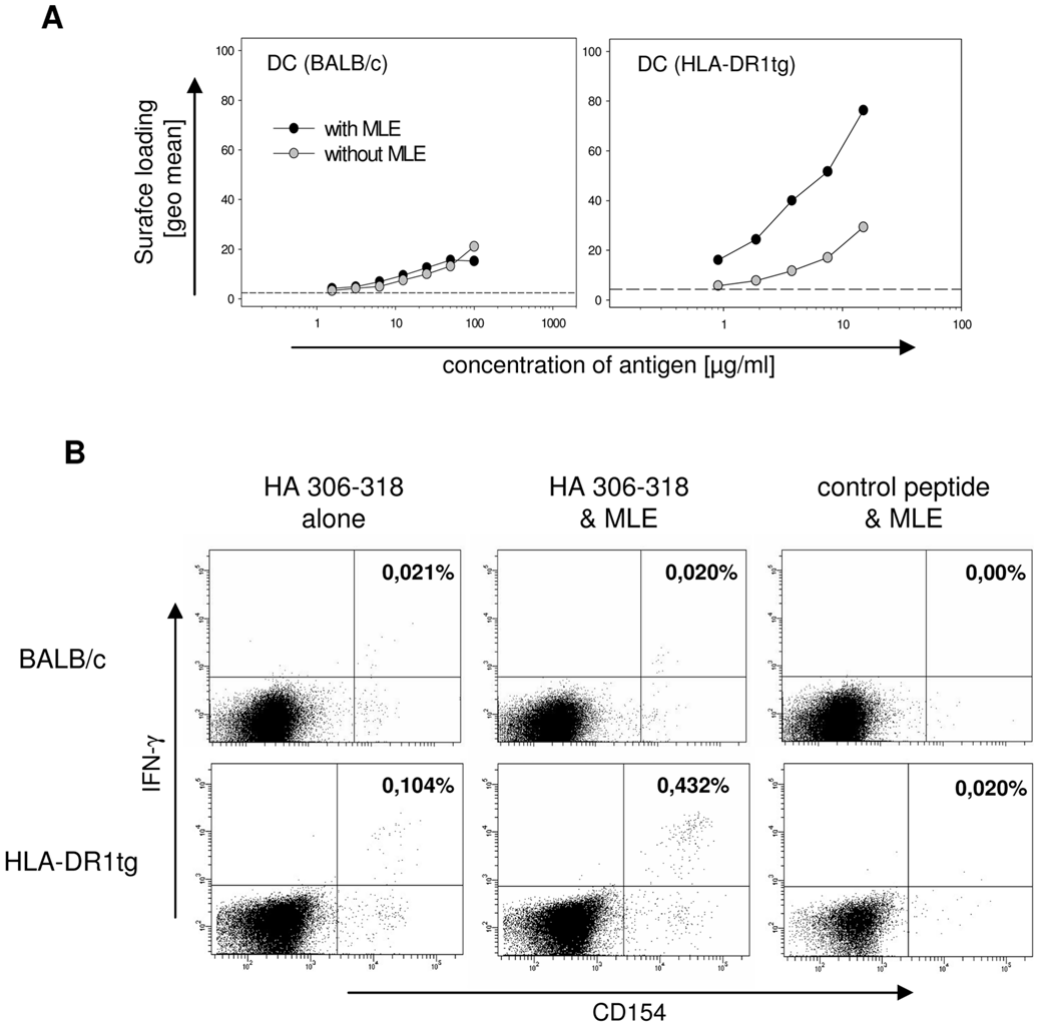
Selective enhancement of CD4+ T cell responses *in vivo* by MLE compounds. (A) Cell surface loading of MLE-susceptible and –non-susceptible MHC molecules. DC generated from non-susceptible BALB/c mice (left panel) and AdEtOH-susceptible HLA-DR1tg (right panel) were incubated for 4 h with the indicated amounts of biotinylated HA 306–318 peptide in the absence (open circles) or presence (closed circles) of 250 µM AdEtOH. Peptide loading was determined on CD11c+ cells by analyzing the mean fluorescence of the streptavidin-signal gated on a distinct expression of HLA-DR. Background fluorescence was detected in the absence of biotinylated peptide (dashed line). (B) Peptide priming of mice. BALB/c (upper panel) and DR1tg (lower panel) mice were s.c. primed with 100 µg and 3 µg HA 306–318, respectively in IFA/CpG supplemented without (left panel) or with AdEtOH (middle panel). Specific T cell response was determined by intracellular flow cytometry staining at day 12 after priming. Lymph node cells were incubated for 6 hrs in the presence of 10 µg HA 306–318 or 10 µg MOG 35–55 as irrelevant control peptide (right panel) and αCD28 antibody. 3 µg/ml Brefeldin A was added for the last 2 h. Intracellular IFNγ-production was analyzed on CD4+ CD154+ double positive T cells. Numbers indicate frequency of CD4+ CD154+ IFNγ+ cells among total CD4+ cells. Data representative of at least two independent experiments are shown.

MLE belonging to the class of adamantane derivates are active on all allelic variants of human MHC II molecule HLA-DR containing the β86Gly residue [18] but not on murine MHC II. To explore the *in vivo* activity of MLE during peptide vaccination we therefore compared their effect in transgenic mice expressing the human HLA-DR1 molecule, which is susceptible to adamantyl-mediated catalysis, with BALB/c mice, expressing only MLE-insensitive murine H2^d^ molecules. Both mouse strains, however, are known to respond to HA 306–318 [26]. As shown in Fig. 2B, BM-derived DC from both mice can indeed be loaded with the peptide in a dose-dependent fashion although HLA-DR1tg DC (right panel) seem to present the antigen more efficient than BALB/c-derived DC (left panel). Most importantly, however, strong enhancement of the peptide loading reaction could be observed only for the cells expressing the human HLA-DR1 molecule.

Based on these results, the specific impact of MLE on the antigen-specific CD4+ T cell response *in vivo* was determined in peptide priming experiments. HLA-DR1 transgenic mice and BALB/c were immunized subcutaneously with HA peptide dissolved in ‘Incomplete Freund's Adjuvant’ (IFA) supplemented both with CpG as TLR-agonist and AdEtOH as MLE compound. Lymph node cells were obtained twelve days later and restimulated *in vitro* in presence of HA peptide. Antigen-specific IFNγ production by CD4+ T cells was assessed by intracellular flow cytometry staining combined with anti-CD154 to identify activated antigen-specific IFNγ-secreting CD4+ cells [27] (Fig. 2B). BALB/c mice showed a weak but specific response against the HA antigen (upper panels). No difference in their frequency was observed whether MLE was present during priming or not. Approximately, 0.02% of the CD4+ cells were detected IFNγ+CD154+ in response to HA, while re-stimulation with a control peptide did not induce any double positive cells. In contrast, the fraction of antigen-specific T cells was more than 4-fold increased in HLA-DR1 transgenic mice primed with peptide/MLE compared to mice primed with peptide only (lower panels). Hence, the result was in line with the anticipated MLE-sensitivity of the two strains documented before in *in vitro* loading experiments (compare Fig. 2A). Moreover, the apparent lack of background stimulation in the absence of peptide indicates that at least under these experimental conditions the MLE vaccine additives do not seem to trigger any unspecific T cell activation after restimulation with an irrelevant peptide (Fig. 2B, right panels).

**Figure 3:**
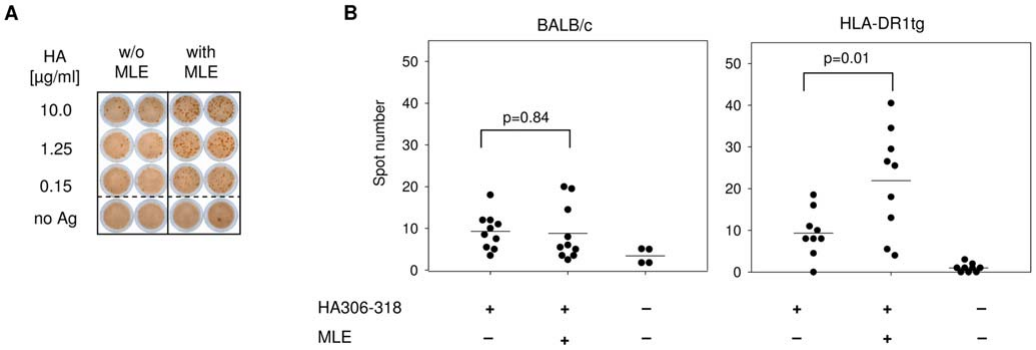
Vaccination in presence of MLE increases the number of antigen-specific IFNγ-producing T cells. (A) Determination of IFNγ in an Elispot assay. 12 days after vaccination, 1×10^6^ lymph node cells from mice primed with 3 µg HA 306–318 in IFA/CpG supplemented without (left panel) or with AdEtOH (right panel) were incubated with titrated amounts of HA306–318 peptide on a plate coated with α-IFNγ antibody. Detection was carried out 48 hrs later by determining the spot number in each well. Spots represent single IFNγ+ cells. (B) Statistical analysis of the T cell response. Summarized Elispot data obtained from groups of BALB/c mice (left panel, n = 10) and HLA-DR1tg mice (right panel, n = 9) were analyzed using *student's t* test.

To further confirm the results obtained *in vivo*, IFNγ secretion of lymphocytes from primed mice was detected by Elispot assay (Fig. 3). Also with this technique a strong increase in the total number of spots, representing single IFNγ producing cells, was detected for HLA-DR1 mice when MLE were added during vaccination (Fig. 3A). The use of larger cohorts of mice allowed us to confirm the above results also statistically. A summary of the data shows that a robust T cell response can be achieved in both BALB/c and HLA-DR1 transgenic mice (Fig. 3B). Notably, only in mice expressing the MLE-susceptible HLA-DR1 molecule a significant increase (p = 0.01) in the frequency of antigen-specific T cells was evident when the priming was carried out in presence of MLE (right panel). MLE had clearly no enhancing effect in MLE-insensitive BALB/c. The average number of spots was even slightly lower, albeit the difference was not significant (p = 0.84). Also no effect on INFγ induction was detected after administration of adjuvant supplemented with MLE alone (data not shown). Thus, the effect is specific and on MLE-susceptible MHC II directly translates into an improved T cell response *in vivo*.

### MLE enhance the CD4+ T cell response against NY-ESO-1 epitopes

**Figure 4:**
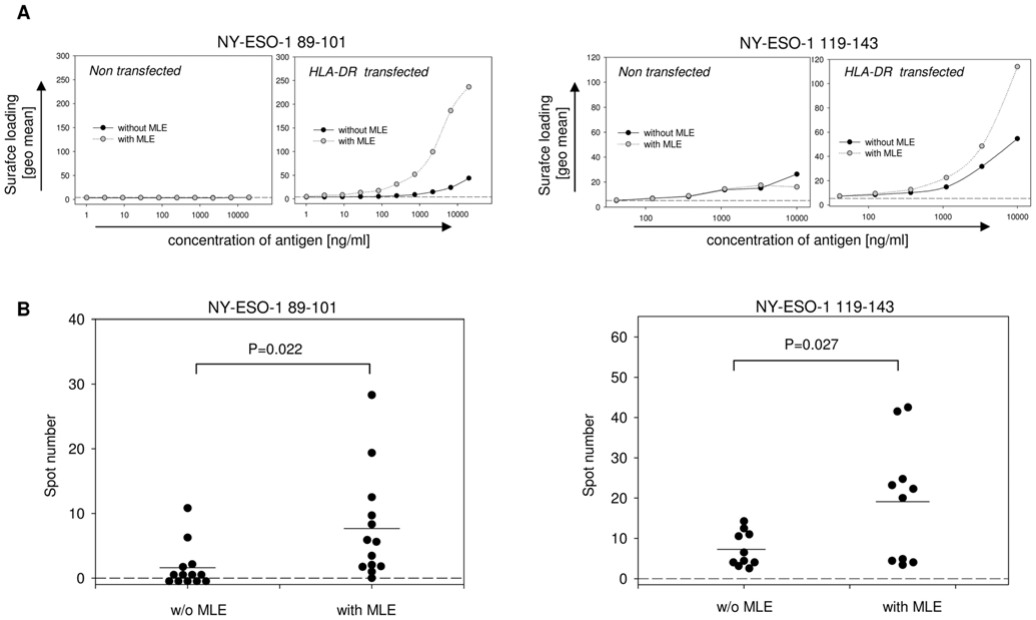
MLE enhances the T cell response against NY-ESO-1 derived epitopes. (A) Cell surface loading of NY-ESO-1 epitopes. L929 fibroblasts transfected with HLA-DR1 (left panel) or HLA-DR4 (right panel) were incubated for 4 h with titrated amounts of NY-ESO-1 89–101 or NY-ESO-1 119–143, respectively. Loading was performed in the absence (closed circles) or presence (open circles) of 250 µM AdEtOH. Non-transfected L929 cells were used as a negative control (left side). Peptide loading was determined by analyzing the mean fluorescence of the streptavidin-signal gated on a distinct expression of HLA-DR. Background fluorescence was detected in the absence of biotinylated peptide (dashed line). (B) Detection of tumour-specific T cell response *in vivo*. Groups of HLA-DR1tg (left panel, n = 13) or HLA-DR4tg (right panel, n = 10) mice were s.c. primed with 5 µg of the respective NY-ESO-1 epitopes in IFA/CpG supplemented with or without AdEtOH. 12 days after vaccination, 1×10^6^ Lymph node cells were incubated with titrated amounts of NY-ESO-1 89–101 or NY-ESO-1 119–143, respectively. IFNγ-detection was carried out 48 hrs later using an Elispot assay and summarized data were analyzed using *student's t* test.

Multiple NY-ESO-1-derived epitopes have been reported capable of provoking CD4+ T cell reponses [28]–[30]. As various forms of cancer cells express NY-ESO-1, these epitopes may therefore represent promising candidates for cancer vaccines. Among those were NY-ESO-1 89–101, which can be presented on HLA-DR1 [31], and the HLA-DR4-restricted NY-ESO-1 119–143 epitope [28], [32]. As both allelic HLA-DR variants are sensitive to adamantyl-mediated ligand-exchange [16], we determined the impact of AdEtOH on these tumour associated antigens (TAA) *in vitro*, using HLA-DR transfected fibroblasts (Fig. 4A). Both epitopes can be loaded onto the surface of cells expressing the relevant HLA-DR molecules whereas no signal is observed in absence of the human MHC class II molecules. Furthermore, adding MLE to the loading procedure leads to a strong increase of NY-ESO-1 89-101 bound to HLA-DR1 (left panels) and to a lower extent also of NY-ESO-1 119–143 bound to HLA-DR4 (right panels).

To determine whether the effect was evident also *in vivo*, HLA-DR transgenic mice were primed with the two NY-ESO-1 epitopes according to the protocol used before for the HA antigen. As shown in Fig. 4B, the addition of MLE to the vaccine revealed again a significant effect on the number of IFNγ-secreting cells. Based on the Elispot assay only 4 out of 13 HLA-DR1 transgenic mice primed with NY-ESO-1 89–101 alone responded to the peptide (left panel). In contrast 12 out of 13 mice reacted against the antigen when MLE was added to the adjuvant and also the average number of spots indicative for IFNγ-secreting cells was significantly increased (p = 0.022). The same applied also for HLA-DR4 transgenic mice primed with the NY-ESO-1 119–143 epitope (right panel) where in the presence of MLE a significant boost of the antigen-specific priming was observed (p = 0.027).

### Enhancement of the immune response against NY-ESO-1 protein

**Figure 5:**
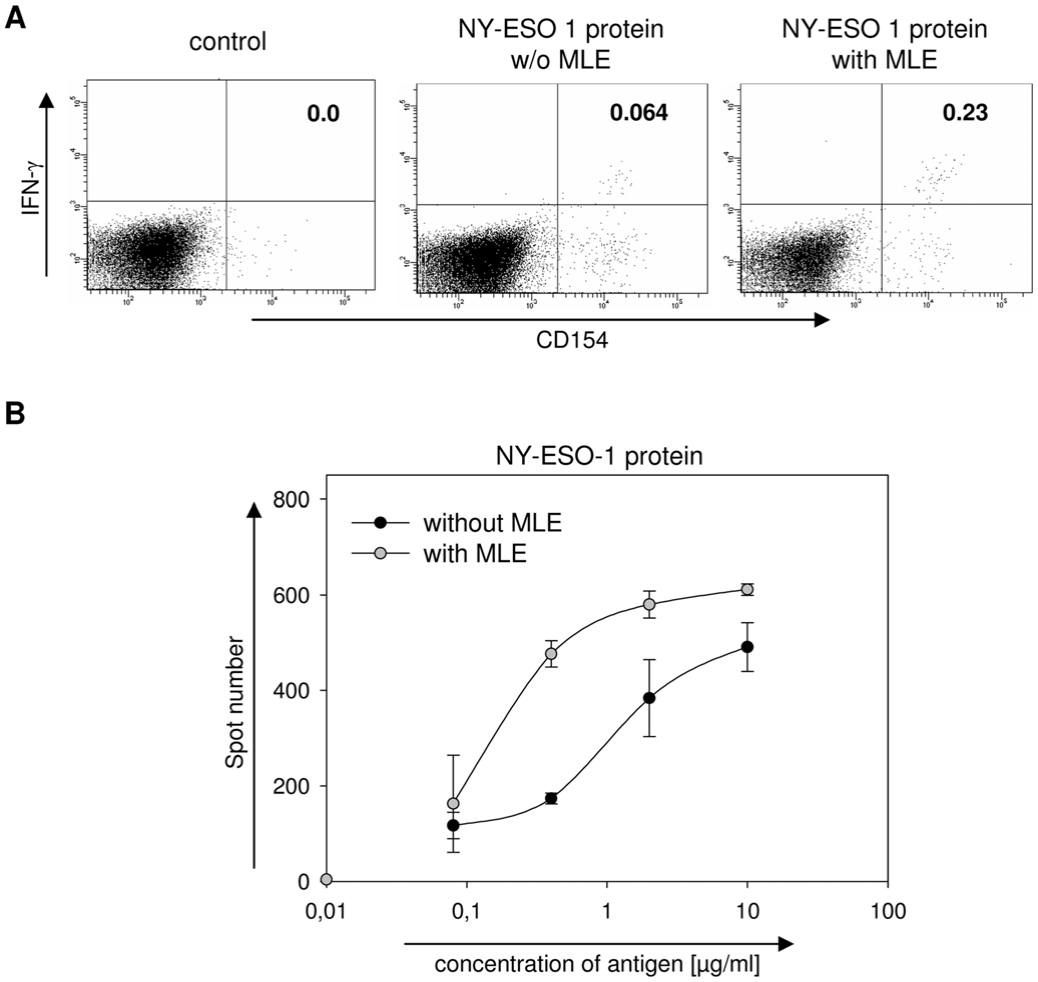
MLE enhance the T cell response against NY-ESO-1 protein. (A) Determination of T cell response by intracellular cytokine staining. HLA-DR1tg mice were s.c. primed with 10 µg NY-ESO-1 protein in IFA/CpG supplemented with (right panel) or without AdEtOH (middle panel). The specific T cell response was determined by intracellular flow cytometry staining 12 days after priming. Lymph node cells were restimulated for 6 h in the presence of 10 µg NY-ESO-1 protein or control peptide (left panel) together with αCD28 antibody. 3 µg/ml Brefeldin A was added for the last 2 h. Intracellular IFNγ-production was analyzed on CD4+ CD154+ double positive T cells. Numbers indicate frequency of CD4+ CD154+ IFNγ+ cells among total CD4+ cells. Data is representative of two independent experiments. (B) Dose-response. Groups of 5 mice were primed with 10 µg NY-ESO-1 protein emulsified in IFA/CpG supplemented with (open circle) or without AdEtOH (closed circle). 12 days after vaccination 1×10^6^ Lymph node cells were *ex vivo* challenged with titrated amounts of NY-ESO-1 protein and IFNγ-detection was carried out 48 hrs later using an Elispot assay. Spot numbers represent single IFNγ+ cells.

Recombinant NY-ESO-1 is a medium sized protein with a molecular weight of ∼23 kD which has been used in multiple clinical trials (http://clinicaltrials.gov). In a previous study we had shown that MLE can also enhance the loading of larger protein fragments onto MHC molecules [15]. We therefore tested whether the effect of AdEtOH was evident also when using the recombinant NY-ESO-1 protein. HLA-DR1 transgenic mice were primed as described before, except that the recombinant protein was used instead of peptide antigens. As shown in Fig. 5A, the single cell analysis of lymph node cells challenged *ex vivo* with the protein 12 days after priming revealed a similar picture as observed for the peptides. Also here, the number of IFNγ+CD154+ CD4+ cells was increased when MLE was present during the priming. The flow cytometry data was further confirmed by a second series of experiments where the response was analyzed in an IFNγ-Elispot (Fig. 5B). In a dose-response study the sensitivity of the NY-ESO-1 specific immune-response increased by almost one order of magnitude. Thus, MLE may be used as additive for both peptide- and protein-vaccines.

## Discussion

So far, only two adjuvant components are regularly employed in human vaccinations: mineral salts or oily emulsifiers as vehicles to form an antigen depot and ‘immune-stimulants’, usually derived from pathogen- or danger-associated compounds that act as agonists for toll-like- (TLR) or other innate receptors [33]. In this study we introduced with ‘MHC-loading enhancers’ (MLE) another class of vaccine additive that improves CD4+ T cell responses by mediating efficient antigen loading of the APC.

Surface MHC II molecules of APC are either blocked by high-affine peptide-ligands or have acquired a non-receptive state [21]. MLE, such as adamantane ethanol (AdEtOH), are able to recover non-receptive MHC II molecules and ‘open up’ free binding sites by releasing ligands with lower affinity [15], [16]. This allows to significantly increase the number of antigen/MHC II complexes on the cell surface of APC, shown here on BM-derived DC, where the strong increase directly translated into a decreased concentration threshold required for T cell activation. *In vivo*, the addition of MLE to the priming mixture resulted in a significantly higher number of activated antigen-specific IFNγ-secreting CD4+ T cells. Also other types of CD4+ effector/memory cells such as Th2 or Th17 cells can be affected in a similar way. In any case, the increase in priming efficiency was specific and apparently based on the interaction of the MLE with the MHC II molecule, since it was evident only in mouse strains expressing MLE-susceptible HLA-DR molecules.

Several classes of compounds have already been identified exhibiting MLE activity. Besides organic structures such as AdEtOH [18] or complex polycyclic compounds [17], [34] also simple aromatic chemicals like p-chlorophenol [15], [16] short peptide derivatives [19] and even certain solvents such as ethanol and n-propanol [20] show the effect. Common to all of them is that they act in a catalytic way independent of the antigen. They can simply be added to the desired adjuvant and can be applied to most of the common protocols used for vaccination.

Up to now, most of the tumour vaccines reported were targeting mainly MHC class I. A number of recent studies confirmed, however, that immunizations addressing CD4+ T cells are more efficient than peptide vaccinations targeting only CD8+ cells [35], [36]. This may apply in particular also for tumour interventions. Considering that tumour-associated T cell epitopes by definition represent self-antigens, they have to be recognized by autoreactive T cells. Their activation, however, is hindered by several safety mechanisms ensuring that tolerance against self is maintained. They are controlled by active suppressor mechanisms [37], [38] and usually express only TCR with a low avidity to their antigen [39]. Successful vaccination therefore needs to overcome tolerance mechanisms to induce a powerful and sustained T cell response. At least in autoimmune diseases the immune response is mostly driven by autoreactive CD4+ T cell responses. Addressing them may therefore be a key for the induction of successful anti-tumour immune interventions, a process that could be aided by MLE.

As shown before [15] MLE catalyse not only the loading of MHC molecules with small peptides, they can also mediate the transfer of larger polypeptide fragments. In this study, a significant enhancement was observed when AdEtOH was added to the recombinant NY-ESO-1 protein used before in clinical trials. While this may suggest its use in protein vaccines, its ability to catalyse the loading of MHC with free protein fragments may also impose a risk. MLE-mediated ligand exchange depends largely only on the availability and we have shown that abundant pathogenic autoantigens, such as multiple sclerosis-associated myelin basic protein (MBP), can be transferred by this mechanism onto MHC II [15]. In fact, Call et al. have shown that the coapplication of encephalitogenic epitopes with chemical MLE-compounds into HLA-DR2 transgenic mice leads to improved peptide loading in vivo [34]. Our own studies further indicate, that the presence of MLE increase the severity of experimental autoimmune encephalomyelitis (EAE) triggered by this antigen (Dickhaut et al., unpublished). Another drawback preventing immediate applications may be the high concentration of MLE used in this study. AdEtOH has to be regarded as a ‘first generation compound’ and further studies are needed to identify improved MLE and to determine their safety in preclinical trials. As a ‘proof of concept’, however, we could show with AdEtOH as an ‘MHC-loading enhancer’ prototype a promising new approach to boost vaccination efficacy at the level of antigen-loading.

## Methods

### Peptides, reagents and antibodies

CpG OND 1826 (5′-tccatgacgttcctgacgtt-3′) was obtained from (Biotez GmbH). Incomplete Freund's adjuvant (IFA) was obtained from Sigma. The following peptides were used: HA 306-318 (PKYVKQNTLKLAT), NY-ESO-1 89-101 (EFYLAMPFATPME), NY-ESO-1 119-143 (PGVLLKEFTVSGNILTIRLTAADHR) and MOG 35–55 (MEVGWYRSPFSRVVHLYRNGK). N-terminal biotinylation was introduced following two 6-aminohexanoic acid spacer residues. Peptides were dissolved in PBS at a stock solution of 1 mg/ml. Recombinant NY-ESO-1 protein was kindly provided by Dr. Gerd Ritter. MLE stocks were kept as a 100 mM 2-(1-adamantyl)ethanol (Sigma) stock solution dissolved in DMSO at −20°C. For flow cytometry staining the following were used: α-CD4-PerCP-Cy5.5, α-CD11c-APC (both from BD PharMingen) α-CD154-PE, α-IFNγ-APC (both from Miltenyi), α-HLA-DR-PE (BD Bioscience), α-CD86-Biotin (produced in our laboratory, clone GL1), streptavidin-APC (Caltag). For Elispot assays the α-IFNγ capture antibody (clone AN18.1724) and the biotinylated α-IFNγ detection antibody (clone R4-6A2) were used, both produced from hybridoma supernatant in our laboratory.

### Animals

Mice were housed in the animal facility of the MDC under SPF conditions and handled in accordance with the institutional guidelines. Abb/HLA-DR4 (Taconic Farms, Denmark) and HLA-DR1 [40] were bred at the MDC facility; BALB/c mice were purchased from Charles River Laboratories (Sulzfeld, Germany) and used at a age 8 to 15 weeks. Abb/HLA-DR4 mice were generated in I-E −/− C57Bl/6, HLA-DR1tg mice are on a B10M/J background. All animal experiments were approved by the Landesamt für Arbeitsschutz, Gesundheitsschutz und Technische Sicherheit (Berlin, Germany).

### Cells

Cells were maintained at 37°C in RPMI/5% FCS. The following cells were used: L929 fibroblasts (ATCC) transfected with HLA-DR1 (DRB1*0101) referred as L57.23 or HLA-DR4 (DRB1*0401) named L243.6, respectively (both provided by E. Rosloniec). HA 306–318 specific HLA-DR1-restricted mouse T cell hybridoma line EvHA/X5 [18]. HA 306–318 specific HLA-DR4-restricted mouse T cell hybridoma line 8475/94 (provided by L. Fugger). Dendritic cells were generated from bone marrow of BALB/c and HLA-DR transgenic mice according to published protocols [41] and cultured in the presence of 10 ng/ml GM-CSF. Maturation was induced on day seven of culture by adding 10 ng/ml LPS (Sigma) for 24 hrs. Maturity was determined by expression of MHC class II and CD86 by flow cytometry analysis. Flow cytometry was performed on a FACSCalibur or LSR II (both BD Bioscience), data was analysed using CellQuest Software (BD Bioscience).

### Peptide-loading of cell surface MHC molecules

Loading experiments were carried out as described [18]. Briefly, 1×10^5^ class II MHC expressing cells per well were incubated in a 96-Well plate with the indicated amounts of biotinylated peptide at 37°C in DMEM supplemented with 5% FCS. MLE were present during the loading reaction at indicated concentrations. After 4 hrs cells were washed and double stained with streptavidin-APC and α-HLA-DR-PE (in PBS/2% FCS) for 20 min at 4°C before being washed and analyzed on a Flow cytometer. Peptide loading was analyzed by determining the SA-APC signal on cells gated on a defined HLA-DR expression. For loading experiments with BM-derived DC cells were pregated on CD11c+ cells for further analysis.

### T cell assay

T cell assays were carried out as described [18]. Briefly, 0,5–1×10^4^ HLA-DR expressing DC per well were loaded with indicated amounts of peptides at 37°C in absence or presence of MLE. After 4 hrs cells were washed and 5×10^4^ HA specific hybridome T cells were added to the culture for 24 hrs in 96 U-bottom plates. T cell response was determined by measuring IL-2 production in a secondary assay using CTL-L cells (ATCC) as described before [20].

### Priming of mice

Mice were primed with indicated amounts of peptide or protein in Incomplete Freund's adjuvant (Sigma) supplemented with 50 µg CpG OND 1826 (Biotez GmbH). MLE were added to a final concentration of 10 mM dissolved directly in Incomplete Freund's adjuvant. Mice were primed subcutaneously on the flanks and neck, injecting a total volume of 200 µl. On day 12, lymph nodes cells and spleens were harvested for *ex vivo* analysis.

### Detection of ex vivo response by Elispot

1×10^6^ freshly isolated Lymph node cells per well were incubated for 48 hrs (37°C, 5% CO_2_, RPMI 5% FCS) with titrated amounts of peptide in Elispot plates (Multiscreen plates HTS 96 well Filtration plate; Millipore) previously coated with α-IFNγ capture antibody (clone AN18.1724). Detection was carried out according to the manufacturer's recommendations, using biotinylated α-IFNγ detection antibody (clone R4-6A2) followed by avidine–HRP enzyme conjugate (Sigma) and 3,3- diaminobenzidine tablets (Sigma). Spots were counted using ‘ImmunoSpot’ reader (C.T.L. Europe GmbH). Samples were set up in duplicates and experiments were carried out at least twice.

### Detection of ex vivo response by intracellular flow cytometry staining

Briefly, 5×10^5^ lymph node cells per well were activated in the presence of 10 µg/ml peptide and 2 µg/ml α-CD28 antibody (produced in our laboratory, clone 37.51) for 6 hrs. Brefeldin A (Sigma) was added for the final 2 hrs at a concentration of 3 µg/ml. Irrelevant peptide together with α-CD28 was used as a negative control. After washing, cell surface was stained with α-CD4 (PBS/2% FCS, 20 min, 4°C) followed by fixation and permeabilization, using the Cytofix/Cytoperm™ Kit (BD Bioscience). Intracellular staining of CD154 and IFNγ was carried out in permeabilization buffer (PBS/2% FCS/0,5% saponin). Antigen-specific cytokine production was assessed by determining the frequency of CD154+ IFNγ+ double positive cells among total CD4+ lymphocytes.
